# Supplementary material for: Are carbon emissions trading and green financial instruments synergistic? -Comprehensive quantitative research based on content analysis
Source: PLoS One. 2024 Mar 7;19(3):e0298601. doi: 10.1371/journal.pone.0298601 (PMC10919601; doi:10.1371/journal.pone.0298601)
Supplement: S1 Data — (ZIP) [file pone.0298601.s001.zip › Data/地级市Z_F工作报告.zip!/─┌├╔╣┼/║⌠┬╫▒┤╢√╩╨/└┤╫╘╣1⁄2╓┌║┼í╛┬φ┐╦╩2╛▌═°í┐_2008.docx]

各位代表：

现在，我代表呼伦贝尔市人民政府向大会作工作报告，请予审议，并请市政协委员和列席人员提出意见。

一、本届政府工作简要回顾

过去的五年，是呼伦贝尔撤盟设市的第一个五年，是呼伦贝尔发展历程发生重大转折的五年。面对艰巨繁重的改革发展任务，市政府在自治区党委、政府和市委的正确领导下，团结带领全市各族人民，以邓小平理论和“三个代表”重要思想为指导，全面贯彻落实科学发展观，有效解决了经济转型、体制转轨带来的诸多困难，成功战胜了非典疫情、禽流感、重大干旱、森林火灾等各种自然灾害，胜利完成了市一届一次人民代表大会确定的各项目标任务。

——综合经济实力大幅攀升。五年来，全市经济呈现出持续快速增长的良好态势。地区生产总值连续跨越了300和400亿元两大台阶，2007年预计达到487亿元，是2002年的2.4倍，年均增长19.1%，人均生产总值达到2452美元。三次产业比例由28.4：24.5：47.1调整为25.5：32.6：41.9。工业总量迅速扩张，在三次产业中的比重不断提升，全部工业增加值达到132亿元，年均增长30％。全面落实各项惠农政策，大力优化农牧业结构，全面启动了新农牧林区建设，着力推进农牧业产业化进程，累计退耕257.6万亩，退牧2025万亩，粮食产量累计完成278亿斤，牧业年度牲畜存栏达到1546.4万头只，农区畜牧业占全市畜牧业的比重五年提高了25.8个百分点。初步形成了龙头带基地、基地连农户的产业化发展格局。林区替代产业长足发展，地方林业职工年均收入达到11000元，森工企业职工年均收入达到10550元。新型边境区域合作不断深入，外贸进出口总额累计完成75.68亿美元，年均增长15%，连续5年位居全区第一。旅游业得到了大发展，2007年，旅游总收入65亿元，比2002年增长49亿元，成为新的经济增长点。全社会固定资产投资累计完成927亿元，年均增长33%，总量超过前九个五年计划投资的总和，为全市经济发展注入了强大动力。财政收入大幅度增长，五年累计完成206亿元，2007年达到70亿元，比2002年增长50.3亿元；五年累计支出330亿元，2007年达到96亿元，比2002年增长了56.7亿元，有力保证了社会事业和社会保障等重点公共支出。金融、保险、邮政等行业均保持良好发展势头。五年来，我们先后获得了“中国优秀旅游城市”、“中国最佳民族风情魅力城市”、“全区民族团结进步先进集体”、“全区社会治安综合治理工作先进地区”等多项殊荣。呼伦贝尔的综合经济实力和地区影响力得到极大提升。

——改革开放取得重大突破。企业改革步伐不断加快，国有、集体中小企业“双退出”改革稳步实施，宝煤、雁煤、扎煤等一批重点骨干企业成功实现资产重组，农垦企业集团化改革顺利实施，国有企业剥离办社会扎实推进，粮食流通体制改革圆满完成。农村牧区综合改革有序开展，全部免除了农牧业税。投融资、财税体制改革取得实效，公共财政体系逐步健全。行政管理体制改革成效显著，行政审批事项精简比例达到48.7%，保留事项全部进入了行政审批中心。紧紧抓住对俄蒙开放战略升级的机遇，口岸基础设施建设、境外资源开发和进口产品落地加工等方面取得重大进展。主动融入东北振兴，积极承接环渤海经济圈、“长三角”、“珠三角”等发达地区产业转移，要素流入势头和经济的外向度不断增强，实现了借力发展。累计引资399亿元，年均增长48.3％；实际利用外资10588万美元，年均增长46.18％。全球500强企业已有3家落户呼伦贝尔。

——基础设施条件明显改善。城市建设坚持“科学规划、合理布局、完善功能”的原则，突出中心城市功能和品位的提升，城市综合承载力和辐射带动力进一步增强。以海拉尔、满洲里为代表的各地中心城镇面貌日新月异，小城镇建设步入自治区前列。全面加大城市基础设施建设力度，累计投入65.8亿元，实施了一大批城镇给排水、垃圾处理、集中供热、市政道路、广场绿化、出口路改造等建设项目。全市累计开发城镇住房面积733万平方米，人居环境得到明显改善。立足“改造提升老城区、加快建设新城区”， 高起点、高标准规划建设了市中心城新区。公路建设累计投资116亿元，实现了旗市区全部通油路，结束了呼伦贝尔没有高等级公路和边远牧业旗不通油路的历史，实现了全部乡镇苏木、90%的村嘎查通公路。铁路建设开工了伊敏—伊尔施铁路，滨洲铁路海满段复线及博克图至兴安岭线路改造工程建成通车，结束了海满段一百余年单线运行的历史。实施了海拉尔民航机场改扩建工程，开通了满洲里民航机场并实施了机场改扩建工程，恢复了停飞10年的海拉尔—赤塔国际航线，开通了国内多条航线，畅通了空中运输渠道。全市初步形成了公路、铁路、航空立体运输格局。以电信传输服务为主的信息产业快速发展，全市邮电业务总量完成11亿元，年均增长18.7%。国民经济和社会信息化取得新进展，农村牧区信息化综合服务取得了阶段性成果。水利基础设施建设累计投入79.5亿元，是“九五”时期投资的17倍，圆满完成了尼尔基水利枢纽工程建设，开工建设了红花尔基水库、农田水利和堤防等工程。全面加强了生态环境保护与建设，海拉尔河、雅鲁河重点流域污染综合治理工作取得重大进展，水体水质基本达到环境功能区划标准。工业污染源治理成效显著，主要城镇空气优良及以上天数达95％。累计投资19亿元，实施了防沙治沙、草原保护与建设、退耕还林还草、天然林保护、重点公益林保护等重点工程，草原退化、沙化得到有效遏制。大兴安岭林区坚持生态优先的原则，强化森林管护，取得了森林资源蓄积、面积双增长的突出业绩。全市进入了国家级生态示范区行列，呼伦贝尔草原被评为“中国最美的草原”。

——各项社会事业长足进步。深入实施科教兴市战略，全面开展了创建全国科技进步先进市活动，启动了科技特派员行动，科技成果向现实生产力转化进程加快，获自治区级科技进步奖21项。优先发展教育事业，圆满完成了“两基”达标的历史任务，高水平实现了民族教育“两主一公”的办学模式，呼伦贝尔学院专升本，结束了我市没有本科院校的历史。大力实施人才强市战略，人力资源开发和人才队伍建设取得新进展。民族文化大市建设蓬勃开展，新闻出版、文学艺术、体育事业、旅游文化节庆活动繁荣活跃，实施了广播电视“西新工程”、“村村通工程”和地方节目传输覆盖工程，广播电视覆盖率显著提高，广大人民群众精神文化生活更加丰富。公共卫生设施建设进一步加强，疾病预防控制、医疗救治和卫生监督水平不断提高，新型农村牧区合作医疗全面展开，参合率达到86%。人口与计划生育工作成绩突出，低生育水平得以稳定。圆满完成了自治区成立60周年庆典我们承担的各项任务。双拥工作成效显著,呼伦贝尔市、满洲里市被命名为“全国双拥模范城”。安全生产工作连续3年受到自治区的表彰奖励。统计、民族、宗教、人防、侨务、气象、档案史志、地震、残联、工商联、红十字会等事业都取得了新的进步。

——人民生活水平显著提高。2007年，预计全市城镇居民人均可支配收入达到10320元，比2002年增长4679元；农牧民人均纯收入达到4520元，比2002年增长2242元。预计社会消费品零售总额达到150.5亿元，年均增长17.1％。居民消费结构加快升级，需求不断扩大，文化、休闲、旅游等消费成为时尚。就业工作成效显著，城镇累计新增就业14.5万人，城镇登记失业率控制在4.4%以内。社会保障体系日趋完善，社会保险覆盖面不断扩大，保障水平显著提高，城乡困难群体救助体系逐步建立。扶贫开发成效明显，累计解决了62.1万贫困人口的温饱问题。投入资金9390万元，改造了“三少”民族危草房6085户，以“三少”民族为主的少数民族群众生产生活条件得到明显改善。

——民主法制建设不断加强。坚持依法行政，决策的科学化和民主化程度不断提高。自觉接受人大依法监督、政协民主监督和各民主党派、无党派人士及社会各界的监督。充分尊重人大代表、政协委员的意见和建议，五年累计办理人大代表议案9件，意见、批评和建议717件，政协委员提案1037件。完善与工会的联席会议制度，重视发挥共青团、妇联等群众团体的积极作用。深入开展政务、厂务和村务公开，扩大了人民群众的知情权和参与权。强化法制意识，行政执法、公正司法水平逐步提高。全面开展“安全稳定呼伦贝尔”创建活动，构建全方位的社会治安防控体系，人民群众的安全感日益增强。信访工作进一步加强，齐抓共管的大信访格局基本形成，信访形势发生了积极变化。加快政府职能转变，政府宏观调控能力、统筹发展能力、公共服务能力和应急处理能力进一步增强。建立健全政风行风评议制度，综合运用审计、监察等监督手段，大力加强行风和廉政建设，投资发展环境不断优化。可以说，过去的五年，是全面建设小康社会取得丰硕成果的五年，是城乡面貌变化最为显著的五年，是各族人民群众得到实惠越来越多的五年，是为呼伦贝尔实现科学崛起奠定坚实基础的五年。

2007年是本届政府任期的最后一年。我们以科学发展观为统领，全面贯彻落实市二次党代会精神，坚持生态立市，高度关注民生，以项目建设为突破口，统筹推进各项工作，经济社会呈现出了新发展、新变化和新进步。一是创建国家级生态市工作全面展开。加大了各项生态保护与建设工程推进力度，采取“退牧、退耕、退伐、退小”等措施，有效地保护了生态环境。认真落实环境影响评价和“三同时”制度，加大节能减排政策的执行力度，对重点流域水污染和重点城市大气污染进行了专项治理，城市环境得到了整体提升。二是坚持点状布局、集中发展，一些大项目纷纷落地。伊敏、宝日希勒两个千万吨级大型露天煤矿建设速度不断加快，灵东矿年产500万吨大型井工矿已经落地，大雁三矿大井顺利实现达产。东能化工40万吨甲醇、新大洲20万吨电石落地开工，金骄生物化工已基本完工，鲁能、大唐、金新等接续化工项目已完成施工准备。80万千瓦城市热电工程通过国家发改委核准，360万千瓦外送电源也将通过批复。乌奴克吐山大型铜钼矿及谢尔塔拉铁锌矿开发已经全面启动。红花尔基水库、两伊铁路等一批基础设施项目建设顺利。扎阿铁路、绥满高速公路前期工作取得了新进展。三是农牧民在结构调整中大幅增收。针对市场变化，引导农牧民调整种养结构取得了明显成效，全市绿色、特色种养业发展迅速，优质、高产、高效农畜产品比例大幅度提高，农牧民在“瞄准市场种养、调整结构挣钱”中尝到了甜头。四是旅游业迅速升温。随着呼伦贝尔知名度、美誉度的迅速提升和住宿、餐饮、通信、航空、公路等基础设施不断完善，各地进一步加大了旅游景区景点建设力度，精心推出了一系列规模大、质量高的节庆活动，促进了旅游迅速升温。旅游接待达480万人次，同比增长15.3%。五是城市面貌焕然一新。各地在继续加大城市建设力度的过程中，重点进行了城市沿街建筑立面改造和背街巷道治理，有效改变了千城一面的陈旧模式，充分展示了以蒙古族、“三少民族”和俄罗斯族文化为主的具有地方民族特色的建筑风格，切实解决了老百姓出行难的问题，改善了人居环境，提高了城市品位。六是全力推出了三台历史文化剧目。整体推出了呼伦贝尔儿童合唱《五彩传说》、大型历史话剧《拓跋鲜卑》、民族歌舞诗《天边》等独具地方特色的文化名片，极大地彰显了呼伦贝尔深厚的文化底蕴和独特魅力。七是高度关注民生和社会事业。加大了对科教文卫等社会事业的投入力度，预算安排的资金比上年增加了17300万元。政府承诺的为民办10件实事全部兑现。圆满完成了年初确定的各项目标任务。呼伦贝尔已经站在了一个新的历史起点上，谋求新的更大的发展。

回顾过去五年来的工作，我们深感成绩来之不易。这是自治区党委、政府和市委正确领导的结果，是全市各族人民团结奋斗的结果，是几任老领导、离退休老干部和社会各界共同努力的结果。在此，我代表呼伦贝尔市人民政府，向全市广大工人、农牧民、知识分子、干部、解放军指战员、武警官兵和政法干警，向各民主党派、人民团体和各界人士，致以崇高的敬意！向所有关心支持呼伦贝尔在科学发展中崛起的海内外朋友，表示衷心的感谢！

各位代表！五年来，我们之所以能够不断克服前进中的困难，化解发展中的难题，在全面建设小康社会的征程上取得重大成就，胜利完成本届政府既定的各项目标任务，主要得益于做到了“五个坚持”：坚持抓住机遇和发挥优势并重，实现了经济快速发展；坚持美丽与发展双赢，有效地保护了青山绿水；坚持全方位对外开放，增强了发展的动力与活力；坚持以人为本，促进了地区、民族之间协调发展；坚持狠抓工作落实，形成了科学发展的强大合力。

在总结成绩的同时，我们也清醒地认识到，全市经济社会发展中还存在一些突出问题和困难：一是结构性矛盾依然突出。二、三产业发展滞后，非公有制经济增速不快，中小企业成长缓慢。二是发展方式比较粗放。农牧业粗放经营的状况尚未根本转变，工业初级产品多，园区主体作用不明显，产业链条短，资源综合开发利用水平不高，环境保护任务比较艰巨。三是改革开放有待深化。自主创新能力和开放水平不高，尤其是对俄蒙开放的深度不够，三国交界的地缘优势没有得以充分发挥。四是区域、城乡差距较大。城乡二元结构矛盾突出，农牧林区发展相对缓慢，尤其是林业危困局面没有从根本上扭转。五是社会事业发展滞后。就业和社会保障压力大，部分群众生活还很困难，涉及群众切身利益的一些问题没有得到根本解决。六是政府职能转变与形势要求还有一定差距。在部分干部中仍然存在思想解放不够、创新意识不强、抓落实力度不大等问题。这些都是发展中的问题，是前进中的困难，只要我们解放思想、坚定信心、凝聚力量，找准解决问题的切入点和突破口，就一定会在今后工作中逐步得到解决。

二、今后五年政府工作的总体思路和目标任务

今后五年，“机遇前所未有，挑战也前所未有，机遇大于挑战”。党的十七大对全面建设小康社会作出了新的战略部署，国家实施西部大开发、东北振兴、对俄蒙开放战略，自治区支持东部盟市全面振兴，对我市的综合支持力度将会更大；经过多年积累，我市已经具备了又好又快发展的基础和条件，尤其是煤炭、电力、化工、石油、有色金属、进出口产品加工等产业已经搭起大的框架，为今后发展奠定了牢固基础；全市上下奋力拼搏、争先创优的氛围日渐浓厚，实现呼伦贝尔在科学发展中振兴的动力会更加强大。我们在倍加珍视难得机遇的同时，要清醒地认识到面临的深层次矛盾和问题也将相互交织，形成更加复杂的制约因素。国家实施功能区划分，宏观经济政策体现“从紧”，使我们一些资源性项目核准加大了难度，项目落地推进的任务更为艰巨，来自区内外横向竞相发展的压力将会更大。针对这些有利条件和不利因素，我们只有进一步深化对市情的再认识，准确把握阶段性特征，不断调整完善思路，创新推进措施，才能争取主动、赢得发展先机。在今后工作中必须遵循以下“五个原则”：一要建设生态文明，实现美丽与发展双赢。正确处理好发展经济与保护生态的关系，牢固树立资源大市也要厉行节约的意识，按照“有进有退、科学发展”的要求，认真做好生态功能区定位，切实把经济发展、社会进步建立在生态良好、环境优良的基础之上，守护住我们世代赖以生存的“绿色净土”。二要又好又快、好中求快，实现科学发展。加快转变经济发展方式，以新型工业化引领产业结构调整，以农牧林产业化引领农牧林区经济结构调整，以贸易物流和旅游文化产业发展带动服务业结构调整，以城镇化推动生产力布局调整，切实提高资源综合利用水平，努力提高区域经济发展的产业支撑能力，做到好中求快、快中见好。三要高度关注和改善民生，构建和谐社会。坚持执政为民的理念，将更多的财力向公共事业倾斜，最大限度地改变社会事业发展相对滞后的现状。高度重视改善下岗失业、受灾群众等弱势群体的生产生活，让各族人民群众共享改革发展成果。四要借助外力、启动内力，全力推进改革开放。深入研究把握国家宏观政策导向，努力争取上级支持。全面扩大对俄蒙开放，积极吸引发达地区要素流入。进一步深化各项改革，推进体制创新和机制创新，消除影响经济社会发展的各种制约因素，增强发展的动力与活力。五要因地制宜、因族举措，促进各民族共同发展进步。针对我市地域辽阔、民族众多、经济类型多样的现实情况，立足实际，分类指导，重点扶持，整体推进。特别要格外关注人口较少民族的发展问题，采取针对性更强的措施，促进各民族、各地区共同繁荣进步。

今后五年政府工作的总体要求是：深入贯彻落实党的十七大精神，高举中国特色社会主义伟大旗帜，以邓小平理论和“三个代表”重要思想为指导，深入落实科学发展观，走建设生态文明和又好又快发展之路。大力构建集约型能源化工、生态型绿色农畜产品、开放型进出口产品加工基地，强力推进新型工业化、新农牧林区建设、新型边境区域合作、新型服务业发展，再造新的经济基础。高度关注和改善民生，加强社会建设，推进城镇化进程，全面加快小康社会建设步伐，努力把呼伦贝尔打造成为全区新的经济增长极。

今后五年处于力争超额完成“十一五”目标、为“十二五”实现良好开局奠定坚实基础的重要历史阶段。全市经济社会发展的预期目标是：实现一个跨越，经济总量跨越1400亿元大关，年均增长20%；人均生产总值达到7000美元，是2007年的近三倍。促进两个增长：城镇居民人均可支配收入年均增长14%，达到20000元；农牧民人均纯收入年均增长12%，达到8000元。确保三个倍增：财政收入增两倍，达到210亿元，年均增长24.6%；固定资产投资增两倍，达到710亿元，年均增长25%；规模以上工业增加值增五倍，达到550亿元，年均增长37.8%。控制四项指标：单位GDP能耗控制在1.5吨标准煤以内，主要污染物二氧化硫年排放量控制在10万吨以内，化学需氧量年排放量控制在1.8万吨以内，城镇登记失业率控制在4.3%以内。到本届政府任期结束时，我们必将迎来一个生态更加良好、经济更加发达、科技更加进步、文化更加繁荣、法制更加健全、社会更加和谐、人民生活更加殷实的呼伦贝尔！

实现上述目标，今后五年必须从以下十个方面“强力推进”：

（一）强力推进生态文明，增强实现可持续发展的能力。以建设生态文明为统领，以“切实保护好内蒙古这块辽阔草原，保护好大兴安岭这片绿色林海”为己任，加强生态文化和生态文明理念教育，提高各族人民的生态意识，形成人人关注生态、人人保护环境的良好氛围。到2010年，全市一半以上的旗市区达到全国生态县创建标准，其余旗市完成80%以上的创建指标；到2015年，努力把呼伦贝尔建设成为国家级生态市，走进全区生态文明建设的前列。着力实施三大工程：一是实施保护工程。科学制定主体功能区规划，按照优先开发区、重点开发区、限制开发区、禁止开发区的主体功能，合理调整生产力和人口布局，有计划、有步骤地把限制开发区和禁止开发区内的人口向生存条件好的地区转移，在生态保护建设重点地区建立“生态无人区”。切实抓好“退耕、退牧、退伐、退小”四大任务，到2012年，完成退耕500万亩，牧区退牧200万头只，林区采伐量控制在200万立方米左右，关闭污染大、能耗高的“五小”企业。二是实施建设工程。在积极争取上级工程项目资金的同时，加大投入，动员全社会力量参与生态文明建设，建立资源补偿机制，全面推进防沙治沙、天然林保护、生态公益林保护和村屯绿化等各级各类工程建设，持续促进草原沙化逐步好转和森林覆盖率逐年提高。三是实施治理工程。加大环境保护执法力度，严厉查处环境违法案件，坚决遏制过度放牧、毁林毁草开荒、乱采滥伐等行为。加强工业污染源治理，严格执行环境影响评价和“三同时”制度，从源头上控制新的污染和生态破坏。抓好嫩江、额尔古纳两大水系水污染防治，保证所有省界、国界断面水质得到有效改善和提高。强化饮用水源保护，各旗市区集中供水水源地水质达标率达到90％以上。抓好城市大气污染防治，保持旗市区所在城镇大气环境质量优良及以上天数达到95％以上。发展循环经济和环保产业，建设科学合理的资源利用体系，形成符合可持续发展要求的经济结构、生态环境系统和社会管理体系，促进生态文明建设健康发展。

(二)强力推进新型工业化，增强工业经济的辐射带动力。深入研究国家宏观产业政策，结合呼伦贝尔资源条件及产业发展方向，全面优化市域工业产业布局。加大现有存量工业企业的扩能改造、提质升级，不断增强企业自主发展能力。加大择商选资力度，培育新的工业经济增长点。切实抓好工业园区发展，加大软硬件建设投入，明确发展定位，提升产业层次，壮大园区经济实力。到2012年，培育出两个以上超百亿园区，园区和基地工业增加值占全市工业增加值80%以上。全力抓好十大产业集群，再造新的经济基础。煤炭产业：高标准、集约化建设扎赉诺尔、宝日希勒、大雁、伊敏四个区域性大型煤炭基地。电力产业：以360万千瓦外送电源和80万千瓦热电联产项目实施为契机，迅速扩大煤电联营坑口电源装机容量，推进伊敏—沈阳50万伏直流送电通道建设，大力推广风力发电、水力发电等清洁能源，到2012年，实现装机1200万千瓦，其中风电100万千瓦。化工产业：大力推进煤炭就地转化增值和矿产资源综合性深度开发，发展以甲醇、二甲醚、合成氨、尿素、烯烃等产品为主的煤化工和石化、氯碱、生物等化工产业，争取到2012年，达到1000万吨化学量。有色、黑色金属产业：以综合利用“两种资源”为目标，进一步加大勘探开发整合力度，推进铅、锌、铜、钼等有色金属以及钢铁等下游冶炼产业的规模开发，到2012年，形成50万吨的铅锌铜钼冶炼能力。石油产业：全力支持大庆石油公司加大勘探开发力度，扩大采油规模，结合境外开发原油及进口原油的落地条件，争取石油化工产业破题，到2012年，实现地产原油200万吨。绿色食品加工业：以打造绿色食品加工基地和带动农牧民增收为目标，培育引进龙头企业，加大基地建设力度，大力推进鲜奶、玉米、大豆、马铃薯、牛羊肉等农畜产品的规模性深度加工转化。进出口产品加工业：依托口岸优势，推进石油、木材、有色金属等进口资源落地加工，推动机电产品、农副产品和蔬菜等出口加工基地建设实现新突破。非资源产业：依托海拉尔、满洲里、牙克石、扎兰屯等中心城市，搭建国内外市场平台，大力发展机械装备制造、电子信息产品制造、机电、生物、创意等非资源产业，到2012年，全市非资源产业占工业比重达到30%。建材产业：以石灰石资源开发、工业废渣循环利用为重点，做强以水泥、新型墙体材料为代表的建材产业，到2012年，全市水泥生产能力达到800万吨。特色种养加工业：以振兴林区经济为目标，以开发山野菜、黑木耳、野生浆果、中草药加工及狐、貂特色养殖加工为重点，鼓励支持特色产业扩大规模，在深度加工转化、培育特色知名品牌上实现新突破。

（三）强力推进农牧业产业化，增强新农牧林区建设的支撑力。以发展现代农牧林业为目标，围绕特色、绿色、生态，大力发展防灾、减灾农牧业，建立以工促农、以城带乡的长效机制，促进农牧林区经济全面振兴。到2012年，全市第一产业增加值达到197亿元，年均增长8%。粮食产量稳定在100亿斤左右。肉类产量达到40万吨，鲜奶产量达到260万吨，水产品产量达到3.5万吨。销售收入超亿元的农牧业产业化龙头企业达到30家左右，绿色和有机农产品比重超过60%，农区畜牧业在全市畜牧业中的比重达到70%，畜牧业产值在一产中的比重达到50%。一要上层次，大力优化结构。扩大马铃薯、玉米、大麦等高产高效农作物种植面积。大力发展农区畜牧业。牧区畜牧业以增加良种奶牛、肉牛、肉羊等优势畜种为主，做到“增大减小、少养精养”，着力提高品质和效益。加大水产资源的保护与科学利用，提高水产品的产量和质量。实施品牌战略，大做绿色文章，培育壮大呼伦贝尔绿色品牌，提高品牌农畜产品产量和市场占有率。二要强支柱，扶强优势企业。加大对现有农畜产品加工龙头企业的扶持力度，加快农牧业产业化经营步伐，重点抓好鲜奶、玉米、马铃薯、大豆、肉、饲料加工等现有龙头企业发展，拉长产业链。积极引进新的农畜产品加工企业，进一步整合资源、集中优势，形成新的产业链，全面提高龙头企业的市场竞争力和带动力，使农牧户在产业化发展中受益。三要增后劲，壮大产业基地。按照规模化、集约化、标准化生产要求，建设乳、肉、粮油、马铃薯、饲草饲料、木材和特色种养七大产业基地，形成规模效应，不断提高原料供给能力和农牧民的综合收益率。四要建机制，提高组织化程度。按照“民办、民管、民受益”的原则，引导扶持各类农牧民专业合作组织加快发展，提高农牧民的组织化程度。加强服务体系建设，增强抵御市场风险的能力。大力发展订单农牧业，建立龙头企业与农牧民的利益联结机制，积极培育和扶持农村牧区各类经纪人、农产品运销大户和各类流通中介组织，促进公司、基地和农牧户形成“风险共担、利益均沾”的合作格局。五要以点带面，全面加快新农牧林区建设。立足“新”字，按照“布局合理、以点带面、体现特色”的原则，坚持从实际出发，尊重农牧民意愿，合理确定新农牧林区建设模式。大力培育特色经济，找准符合当地实际的发展路子，壮大集体经济实力，千方百计化解镇村两级不良债务。积极拓宽农牧民增收渠道，大力提高生产生活水平。加强德育新风教育，形成健康文明的道德风尚。加强交通、文化、卫生等基础设施建设，整治村屯环境，改善农村面貌。坚持村民自治，推进民主管理。通过五年的不懈努力，使全市农牧林区面貌发生比较大的变化，到2012年，全市50%以上的村嘎查实现经营产业化、生产标准化、农牧林区文明化、管理民主化和农牧民知识化。

（四）强力推进城镇化，增强县域经济发展综合承载力。按照“优化布局、提升功能、适度超前”的原则，发挥城乡规划的前瞻指导作用，抓好城镇规划与产业规划衔接，构建具有林区、牧区、农区和边境城市鲜明特色的城镇体系。到2012年，全市城镇化率达到72%，人均住宅建筑面积达到28平方米以上，人均拥有铺装道路面积14平方米。以海、满、牙、扎等区域性中心城市为重点，建设一批有特色、有产业支撑的城关镇、口岸城镇和旅游城镇，塑造地域民族特色鲜明的城镇风貌。加强中心城平房区改造，加快经济适用住房建设，健全廉租住房制度，抓好城市给排水、垃圾处理、集中供热、市政道路改造、园林绿化等基础设施建设，进一步改善城市人居环境。全面加大呼伦贝尔市中心城建设力度，拉开城市框架，做大城市规模。继续坚持市区共建，把海拉尔打造成呼伦贝尔经济发展核心区。加快公路建设，提高公路等级，到2012年，全市公路建设投入累计达到180亿元，总里程达到1.5万公里。推动铁路主要干线后方通道及延伸建设，“两伊”铁路实现全线贯通，全力促进扎兰屯—阿荣旗、海拉尔—黑山头、室韦—莫尔道嘎、满洲里—新右旗—新左旗—阿尔山等铁路建设。进一步加强海拉尔国际航空港建设，改善航站基础设施条件，拓展国际国内航线。

（五）强力推进改革开放，增强地区发展进步的活力。进一步深化财政体制改革，建立健全公共财政体系，大力培植税源，优化支出结构，促进公共服务均等化。推进投融资体制改革，完善金融体系建设，引入股份制商业银行，加强金融诚信体制建设，树立诚信意识，创造良好的金融环境。深化文化、科技、卫生和事业单位改革。建立国有资本经营预算制度，完善国有资产管理体制。加快农垦、煤炭等国有大企业体制、机制和管理创新步伐，完善现代企业制度。鼓励具有优质资产、优秀人才和优良业绩的企业以资产为纽带进行跨地域联合重组，提高企业的核心竞争力。支持森工集团剥离企业办社会，增强企业发展活力。建立优化经济发展环境的长效机制，着力打造具有政策、资源、产业优势的引资“洼地”，积极利用好东北振兴的各项政策，主动加强与沿海发达地区的交流与合作，多渠道联系国内外知名大企业，引进资金、技术和人才等生产要素，以大开放促进大发展。充分发挥对俄蒙开放中满洲里的龙头作用和相关旗市区的支撑作用，推进满洲里与相关旗市区、大企业的双向互动，全力构建以满洲里为龙头，以黑山头、室韦对俄口岸和阿日哈沙特、额布都格对蒙口岸为“两翼”，以滨洲线为“一带”的“一带两翼”边境区域合作新格局。进一步加强与俄蒙毗邻地区的友好合作，开拓俄蒙市场，扩大贸易往来，调整出口结构，转变外贸增长方式，有条件的旗市区都要“走出去”，促进外向型经济加快发展。到2012年，全市进出口总额年均增长18.2 %，达到60亿美元，直接利用外资9580万美元。

(六)强力推进非公有制经济发展，增强发展的驱动力。毫不动摇地鼓励、支持、引导非公有制经济发展，促进非公有制经济发展壮大，成为经济发展的生力军。到2012年，全市非公有制经济在经济总量中比重和全社会固定资产投资中的比重均达到50%以上。大力优化非公有制经济发展环境，加大政策、信息、资金等扶持力度，在市场准入、投融资、税收、土地使用和对外贸易等方面创造宽松环境。建立健全社会化服务体系，加强对民营企业的协调、服务和指导，加大对中小企业的扶持力度，推动“一个产业带动百户中小企业工程”。鼓励、支持民营企业进入公用事业、基础设施、金融保险、教育、卫生、文化等领域发展。建立高素质的企业家队伍，注重本土企业家队伍培养。着力提升非公有制经济的发展质量与素质，引导民营企业在发展模式、科技进步、融资扩张等方面不断创新，解决布局分散、小规模经营、低水平扩张等突出问题，步入区域化布局、专业化经营、市场化联动、社会化协作、集群化发展轨道，不断增强发展后劲。

（七）强力推进新型服务业，增强经济发展的新动力。全力构建以各旗市区中心城镇为重点的商贸中心，以各大旅游景区为重点的旅游中心，引领新型服务业长足发展。到2012年全市第三产业增加值达到505亿元，年均增长17%。依托我市全生态、原生态的旅游资源优势，针对国内外市场，主打“呼伦贝尔大草原、大冰雪、大森林、北方游牧民族摇篮、蒙古族发祥地、最佳民族风情魅力城市、中俄蒙三国交界”等品牌，优化旅游区域布局，强化区域联动和产业联动，加强形象宣传和旅游促销，采取引进外资和启动内资相结合等措施，全力打造海拉尔民族风情魅力城、满州里国际口岸名城、牙克石冰雪森林之都、扎兰屯风景名胜城等旅游基地。加强旅游基础设施建设，开发呼伦贝尔民族风情园、白音哈达成吉思汗实景演出地、根河敖鲁古雅主题文化园、莫日根河观光牧场、达赉湖度假区等重点景点、景区。依托扎兰屯国家级风景名胜区的品牌优势，加快柴河—阿尔山景区建设，打造岭南休闲度假旅游胜地。进一步完善旅游基础设施建设，全面提升“吃、住、行、游、购、娱”综合承载能力。着力加强行业管理，提升服务质量，树立呼伦贝尔的旅游形象。抓住新一轮经济增长和消费结构升级的机遇，促进面向俄罗斯、蒙古国和国内市场的物流业发展，建设大市场，搞活大流通，打破市场封锁和行业垄断，重点建设、培育、发展一批专业特色鲜明、集散功能较强的物流基地和专业市场。着力推进会展、信息、金融、咨询、法律服务等现代服务业发展。

（八）强力推进科教兴市，增强全面振兴的核心竞争力。全面实施科教兴市战略，深化科技体制改革，激活整合现有科技资源，培育技术创新体系，注重提高自主创新能力，建设创新型呼伦贝尔。继续推进科技特派员行动，加快科技示范园基地建设，加快科技推广步伐，引导和支持创新要素向企业集聚，促进科技成果向现实生产力转化。加快各级各类教育协调发展，基本普及15年规范的国民教育，实现城乡教育均衡发展，提高教育现代化水平。完善职业教育体系，优先发展民族教育，满足人民群众对各级各类教育的需求。支持呼伦贝尔学院完成本科院校评估，创建呼伦贝尔高等职业技术学院。推进社区教育，构建终身教育体系。加大特殊教育投入，提高残疾人受教育水平。坚持“引进、提高、整合”的原则，培育信息产品制造业，提升全市工业、农牧业和社会信息化水平。全面实施人才强市战略，牢固树立“人才资源是第一资源”的观念，深化人事制度改革，加强人才引进、培养、储备和人才市场建设。统筹抓好以高层次人才为重点的各类人才队伍建设，努力形成一支规模较大、素质优良的创新人才队伍，为经济社会全面进步提供有力的人才支撑。

（九）强力推进民族文化名市建设，增强呼伦贝尔的软实力。立足自然、历史、民族、地域等资源优势，依托蒙古族、达斡尔、鄂温克、鄂伦春和俄罗斯族厚重的文化底蕴，努力打造独具魅力的呼伦贝尔民族文化名市。重点实施“五大工程”：一是文化精品打造工程。牢固树立精品意识，切实抓好精品创作，努力打造呼伦贝尔儿童合唱《五彩传说》、民族歌舞诗《天边》、历史话剧《拓跋鲜卑》、实景演出《成吉思汗》、《蒙兀室韦—蒙古古乐》五张文化名片。抓好特色乌兰牧骑建设，鼓励支持文学、歌舞、戏曲等各类文化精品创作，推出一批具有影响力的名著、名剧、名歌、名舞。加强文化交流和精品推广，举办好国际儿童合唱节。二是文化产业开发工程。加强对文化产业发展的宏观指导，优化文化产业发展环境，放宽市场准入条件，引导民营资本进入文化领域，形成文化产业多元投入机制。强化文化市场建设，鼓励发展创意文化产业，把旅游、城建等行业与文化产业发展有机结合起来，加快文化产业化发展进程。三是文化设施建设工程。着力建设一批体现我市文化特色，代表地区形象和发展水平的标志性文化设施，重点完成市博物馆、体育馆、图书馆、档案馆、科技馆等工程建设。健全和完善四级文化设施网络。推进新一轮广播电视“村村通”和无线覆盖工程，加快广播电视数字化进程，提高广播电视节目制作播出能力。四是文化遗产抢救保护利用工程。全面开展对蒙古、鄂伦春、鄂温克、达斡尔和俄罗斯族文化的保护与研究，完成非物质文化遗产和全国第三次文物普查工作，大力推进历史文化名城和城区、乡镇等各级文物保护建设。五是基层群众文化体育工程。深入开展创建文化、体育先进旗市区活动，大力实施“彩虹文化计划”、“边境文化长廊规划”以及“百县千乡宣传文化工程”，举办各类节庆活动，加强体育基础设施建设，广泛开展全民健身运动，吸引广大人民群众积极参与，丰富文化体育生活。在全社会开展道德模范创建评比活动，建立道德模范奖励基金，大力弘扬“存好心、做好人、办好事”的道德风尚，提高全体公民的文明素质。通过全市各族人民共同努力，实现我市文化大繁荣、大发展，为呼伦贝尔全面振兴提供强大的精神动力。

（十）强力推进民生事业，增强构建和谐社会的凝聚力。以大力实施“六个一幸福指数”工程为引领，以改善民生为目标，统筹发展各项社会事业，努力使各族人民学有所教、劳有所得、病有所医、老有所养、住有所居，全力推进和谐社会建设，增强边疆少数民族地区的凝聚力。一要提高干部群众的收入水平。将新增财力更多地用于公共支出，较大幅度地提高国家机关和事业单位人员津贴，提高行政事业单位公务费标准。建立企业职工工资正常增长机制和支付保障机制，落实最低工资标准政策，逐步提高劳动者工资水平。采取切实可行的措施，提高低收入群体和弱势群体的收入水平。做好扶贫开发工作，切实减少贫困人口数量，改善贫困群众生产生活条件。二要提高就业和再就业水平。实施积极的就业政策,加强政府引导，健全市场就业机制，扩大就业规模，改善就业结构，完善就业援助制度，加强农牧林区富余劳动力转移就业培训，及时帮助零就业家庭解决就业困难，积极做好高校毕业生就业工作。到2012年，累计实现城镇新增就业15.5万人，“就业困难对象”再就业2.5万人，农牧林区劳动力外出就业18万人。规范和协调劳动关系，落实国家对农牧民工的各项政策，依法维护劳动者的权益。三要提高各族人民的健康水平。努力建立基本医疗卫生制度，加强农村牧区三级卫生服务网络和城市社区卫生服务体系建设，提高重大疾病防控和突发公共卫生事件应急处置能力。强化食品药品安全监管，实施“万村千乡市场工程”、“食品放心工程”，加强市场管理，改善消费环境，确保人民群众饮食用药安全。积极推进城乡社区建设，完善社区服务，满足人民群众不断增长的生活服务需求。四要提高社会保障水平。进一步完善社会保障体系，提高保障层次和待遇水平，加强社会保险基金征缴、监督和管理，保障人民的基本生活。扩大农村牧区社会养老保险试点范围，建立健全城镇职工、居民基本医疗保险制度，完善农村牧区新型合作医疗制度，开展城市医疗救助，形成多层次医疗保障体系。适当提高城市低保标准，扩大农牧区低保覆盖面。逐步完善城乡社会救助体系，切实维护和保障各类困难群众的基本生活权益。五要提高就学保障水平。认真贯彻落实农村牧区义务教育经费保障机制和“两免一补”政策，建立和完善家庭困难学生资助体系，给予义务教育阶段中小学蒙语授课寄宿生生活补助，推进大学生生源地财政贴息贷款工作，千方百计解决好困难家庭学生就学难的问题。六要提高少数民族群众生产生活水平。认真抓好国家级、自治区级兴边富民重点县各项工作，坚持争取外援和自力更生并重的原则，确定发展重点，强化扶持措施，全力推进以“三少”民族为重点的少数民族地区经济社会快速发展，促进各民族共同发展进步。七要提高安全稳定水平。继续开展“安全稳定呼伦贝尔”创建活动，严厉打击各种刑事犯罪，强化社会治安综合治理。落实安全生产监管责任，全力推进安全生产进乡镇、入社区工作，确保人民生命财产安全。高度重视人民群众来信来访工作，畅通群众的诉求渠道，完善矛盾纠纷调处和信访工作问责机制，积极解决群众关心的热点、难点问题。进一步做好优抚安置工作，深入开展双拥共建，加强军政军民团结，共筑北疆安全屏障。重视老龄工作，落实老干部待遇。加强人口和计划生育工作，提高人口素质，稳定低生育水平。认真做好统计、档案史志、人防、气象、地震、民族、宗教、红十字会等工作。

2008年是全面贯彻落实党的十七大精神的第一年，也是新一届政府的起步之年。2008年全市经济社会发展主要预期指标是：地区生产总值增长18%，达到590亿元。规模以上工业增加值增长28%，达到176亿元。固定资产投资增长35%，达到310亿元。财政收入增长20%，达到84亿元。城镇居民人均可支配收入增长14%，达到11770元。农牧民人均纯收入增长12%，达到5060元。社会消费品零售总额增长15%，达到173亿元。外贸进出口总额增长14%，达到30亿美元。人口自然增长率控制在5.43‰以内。城镇登记失业率控制在4.3%以内。单位GDP能耗控制在1.8吨标准煤。在具体工作中，2008年将为民办10件实事、推进20个重点公共公益项目和10个工业项目。10件实事有：呼伦贝尔中心区城镇居民供水水源改造一期工程，城镇居民社区活动场所建设，智障残疾儿童康复就学场所建设，蒙授高中家庭困难学生扶困助学，城镇居民基本医疗保险，农村牧区饮水安全，低收入家庭廉租住房及经济适用住房建设，城镇就业再就业及特困家庭子女助学，“少少”民族猎民生产生活补助，城市低保家庭燃煤补贴。20个公共公益项目有：海拉尔火车站改造，新城区基础设施建设，满洲里棚户区改造，满洲里新国际货场一期工程，阿荣旗—扎兰屯铁路建设，海拉尔机场路改扩建，甘南—牙克石高速公路，三少民族自治旗通乡公路改造，沙地治理，草原保护和草原生态补偿，节水灌溉推广，职业技术学院一期工程，市蒙医医院，市结核病院住院楼，市图书档案馆，市体育场，鄂伦春自治旗文体活动中心，成吉思汗实景演出，市民族风情园，牙克石凤凰山庄高级滑雪场。10个工业项目有：450万千瓦电源点，驰宏矿业公司年产6万吨铅14万吨锌冶炼，中国黄金集团10万吨铜冶炼，西洋集团俄罗斯别列佐夫铁矿综合开发，新大洲20万吨电石转12万吨PVC，东能化工40万吨甲醇，金新集团年产50万吨合成氨80万吨尿素，大唐集团年产18万吨合成氨30万吨尿素，华泰集团一期年产5万辆汽车，嵯岗石油转换站。这些实事和重点项目事关全市经济社会发展的大局、事关全市各族人民的福祉，全市上下一定要树立一盘棋思想，精心组织，全力推进，确保按期完成。以上项目，在2008年人代会上还将做出具体部署。

三、建设为民、高效、务实、法治、廉洁的服务型政府

新一届政府要在市委的正确领导下，做到依法行政、执政为民。进一步解放思想，充分凝聚各方面的智慧和力量；进一步增强忧患意识，调动各方面的积极性和创造性，努力建设为民、高效、务实、法治、廉洁的服务型政府。

（一）建设执政为民的政府。“执政之基在于民”。要始终保持同人民群众的血肉联系，做到与人民群众同呼吸共命运的立场不能变、全心全意为人民服务的宗旨不能忘、从群众中来到群众中去的路线不能丢，切实把人民群众的利益维护好、实现好、发展好。各级政府、各部门要切实加强执政能力建设，在执行和制定政策过程中，既要大胆解放思想，开拓创新，打破一切阻碍经济社会发展的制约和束缚，促进经济社会又好又快发展；又要树立民生无小事的理念，细心体察，换位思考，常思为民之策，常兴为民之举，常怀为民之心，做到为官一任，造福一方。要建立健全为民办实事的工作机制，形成各级政府每年都要为民切实办一些群众所急、所想的实事好事，全面提高各族人民群众的幸福指数。

（二）建设高效协调的政府。“效能是政府工作的生命”。 要全面提高政府的公信力，继续深化行政审批制度改革，强化行政审批中心的功能，大力优化发展环境，为旗市区、大企业、民营企业和园区提供“全天候、全方位、全身心、全过程”的服务。各级各部门要团结一致，通力合作，上下连动，提高效率。凡是涉及多个部门和多家单位的事项，相关部门和单位都要主动联系、搞好协调，争做政府提速的“加油站”；决不允许出现“中梗阻”和推一推动一动甚至推而不动的现象。切实做到令行禁止，确保政令畅通。要下决心解决个别部门存在的中央政策不会用，区市政策用不活，只讲上级对口部门要求，不看地方实际，死抠条文、生搬硬套，起步不快后退快，启动不灵刹车灵等问题，全面提高政府的行政效能。

（三）建设务实奋进的政府。“空谈误国，实干兴邦”。各级政府要大力弘扬求真务实的工作作风，一切从实际出发，按客观规律办事。既要积极进取，又要量力而行，不盲目攀比；要珍惜民力，不搞劳民伤财的“形象工程”；要察实情，讲真话，不虚报浮夸；要坚持统筹兼顾，立足当前，着眼长远，不急功近利；要厉行节约，健全机制，努力降低行政成本。各级各部门要大力精简会议、减少文件，大力提倡开短会、讲短话、发短文，腾出更多时间和精力，深入基层，调查研究，解决问题。要强化激励约束机制，进一步完善考核奖惩办法，形成以发展论成效、以实绩见高低、以贡献看能力的良好氛围。加大政务公开力度，加强电子政务建设，推行“阳光行政”、打造“透明政府”。在各级政府和各部门形成务求实效、开拓奋进的实干氛围，共同唱响全面振兴的主旋律。

（四）建设民主法制的政府。按照“科学行政、民主行政、依法行政”的要求，积极推进政府管理理念、管理机制和管理方式创新。完善公众参与、专家论证、政府决策相结合的决策机制和重大决策失误责任追究制度。对涉及经济社会发展全局和群众切身利益的重大事项，要通过政府新闻发布会、重大项目听证会等方式，广泛征询意见，保证群众依法行使民主参与和民主监督权利。自觉接受人民代表大会及其常委会的依法监督和人民政协的民主监督，认真听取民主党派、无党派人士及各人民团体意见，接受新闻的舆论监督和公众的社会监督。加强政府系统内部监督，支持监察、审计部门依法独立履行监督职责。各级领导干部要带头维护宪法和法律权威，严格按照法定权限和程序行使权力、履行职责，树立法治政府的良好形象。

（五）建设廉洁自律的政府。“公生明，廉生威”。各级领导干部只有公道正派、廉洁自律，才能真正赢得群众的信任、拥护和爱戴，才能担负起人民赋予的历史重任。要坚决贯彻标本兼治、综合治理、惩防并举、注重预防的反腐倡廉工作方针，推进教育、制度、监督并重的惩治和预防腐败体系建设，认真落实领导干部廉洁自律各项规定，积极开展专项治理和监督检查，严肃查处违纪违法案件，坚决制止损害群众利益的行为。深入开展效能监察，解决不作为和乱作为等问题。各级领导干部要时刻牢记权为民所用、情为民所系、利为民所谋的宗旨，决不能把权力作为谋取私利的工具。要从小事做起，从小节开始，耐得住清贫，淡泊名利；管得住家人，谨慎交友，时刻保持清醒头脑。做到自重、自省、自警、自励，自觉地过好权力关、金钱关、名位关和人情关。努力创造风清气正的工作氛围，塑造为民服务的政府形象。

各位代表：回首过去，我们倍感欣慰；展望未来，我们豪情满怀。呼伦贝尔已经站在了一个新的历史起点上，开启了一个新的创业征程。让我们以崭新的风貌，昂扬的斗志，扎实的工作，认真贯彻落实党的十七大和市委二届八次全委会精神，与时俱进，开拓创新，奋勇争先，为实现呼伦贝尔的全面振兴而努力奋斗！
